# Fecal calprotectin levels in pediatric cow's milk protein allergy

**DOI:** 10.3389/fped.2022.945212

**Published:** 2022-08-09

**Authors:** Dominika Lendvai-Emmert, Vanessza Emmert, Alexandra Makai, Katalin Fusz, Viktória Prémusz, Kata Eklics, Patrícia Sarlós, Péter Tóth, Krisztina Amrein, Gergely Tóth

**Affiliations:** ^1^Doctoral School of Health Sciences, Faculty of Health Sciences, University of Pécs, Pécs, Hungary; ^2^Department of Neurosurgery, Medical School, University of Pécs, Pécs, Hungary; ^3^Neurotrauma Research Group, Szentágothai Research Centre, University of Pécs, Pécs, Hungary; ^4^Erzsébet Teaching Hospital and Rehabilitation Institute, Sopron, Hungary; ^5^Institute of Physiotherapy and Sport Sciences, Faculty of Health Sciences, University of Pécs, Pécs, Hungary; ^6^Department of Physiology, Medical School, University of Pécs, Pécs, Hungary; ^7^Department of Languages for Biomedical Purposes, University of Pécs, Pécs, Hungary; ^8^First Department of Medicine, Medical School, University of Pécs, Pécs, Hungary

**Keywords:** food allergy, children, cow's milk protein allergy, fecal calprotectin, elimination diet, dairy, Gastroenterology

## Abstract

**Introduction:**

The most prevalent food allergy in younger children is cow's milk protein allergy (CMPA), a hypersensitivity reaction to cow's milk protein and its most common clinical manifestation is allergic colitis. The goal of our recent study was to assess somatic symptoms of CMPA and to prospectively observe the effects of a dairy elimination diet using objective parameters and questionnaires.

**Methods:**

The County Hospital in Szekszárd, Hungary, investigated children aged 1 to 18 who had clinical signs that might indicate CMPA. Stool samples were taken and analyzed using a fecal calprotectin (FC) rapid test (Quantum Blue fCAL, Bühlmann Laboratories, Switzerland) at the time of the diagnosis and following 3 months of an elimination diet. At the baseline visit as well as the first and second follow-up, questionnaires were filled out. Patients were divided into two subgroups according to dietary guidelines based on the results of the questionnaires.

**Results:**

A total of 47 patients participated in the study [42.55% female, mean age: 7.36 (SD 4.22) years]. There was no significant difference in FC levels between baseline and after 3-month elimination diet [73.98 (71.12) μg/g and 68.11 (74.4) μg/g, respectively, *p* = 0.331]. After three months, there was a significant decrease in FC levels among patients who adhered to the strict diet [84.06 (79.48) μg/g and 41.11 (34.24) μg/g, respectively, *p* = 0.001].

**Conclusion:**

The findings of our study suggest that FC can be an objective marker in confirming the diagnosis of CMPA. Significant improvement in clinical symptoms and in FC levels can only be expected after a strictly followed elimination diet.

## Introduction

Cow's milk protein allergy (CMPA) is a hypersensitivity reaction to cow's milk protein which can manifest as a variety of clinical symptoms (1). The prevalence of the condition among infants is 2–6% and decreases with age, though it can occur at any age (2–6). The prognosis of CMPA is favorable, according to Polgár, 15% of infants with CMPA show no symptoms at 1 year, 51% at 3 years and 67% at 4 years of diet (3). Host examined the duration of recovery from the introduction of the elimination diet: 45–50% of the patients presented no symptoms after 1 year, 60–75% after 2 years, 85–90% after 3 years, 90–95% after 5–10 years of diet (6–9).

The most common clinical manifestations of CMPA include gastrointestinal, skin and respiratory symptoms, as well as behavioral changes. Allergic colitis is among the most frequent symptoms of CMPA, which may present with abdominal pain, diarrhea and/or hematochezia, bloating and vomiting. The majority of these clinical signs recede rapidly after the introduction of the elimination diet, although remission of allergic colitis cannot be objectively assessed based on our previous findings (10). Detection of calprotectin in stool samples provides information on the presence and severity of allergic inflammation. However, measuring fecal calprotectin (FC) levels in patients with CMPA is not a widely used method. Only a few minor studies have been conducted on the diagnostic and prognostic value of FC measurement in patients with CMPA (11–13).

C has played a major role in the diagnosis and monitoring of gastrointestinal inflammation over the last 25 years. The quantification of the above-mentioned biomarker is a simple, fast, and relatively inexpensive procedure. Calprotectin (also known as MRP8/14 or S1000A8/A9) is a 36 kDa calcium and zinc-binding protein, a member of the S-100 family of proteins that was first isolated from white blood cells. Calprotectin makes up 60% of the cytosolic proteins of neutrophil granulocytes, but it is also present in large amounts in monocytes and macrophages, thus, it is a widely detectable substance in the human body: in blood plasma, urine, cerebrospinal fluid, faces, saliva, and synovial fluids (14–16). It is involved in several physiological processes like being an active mediator of inflammatory processes and enhancing the involvement of neutrophil granulocytes in migration, adhesion, phagocytosis and chemotaxis.

Since FC is in immediate contact with intestinal mucosa, it is a more sensitive marker of intestinal inflammation or other intestinal pathologies than serum markers. This simple, non-invasive and cost-effective test is the most widely used method for detecting and monitoring bowel inflammation (17, 18).

The goal of our recent study is to assess somatic symptoms of CMPA and to prospectively observe the effects of dairy elimination diet on them with questionnaires and objective parameters.

## Methods

### Study design

**Figure 1:**
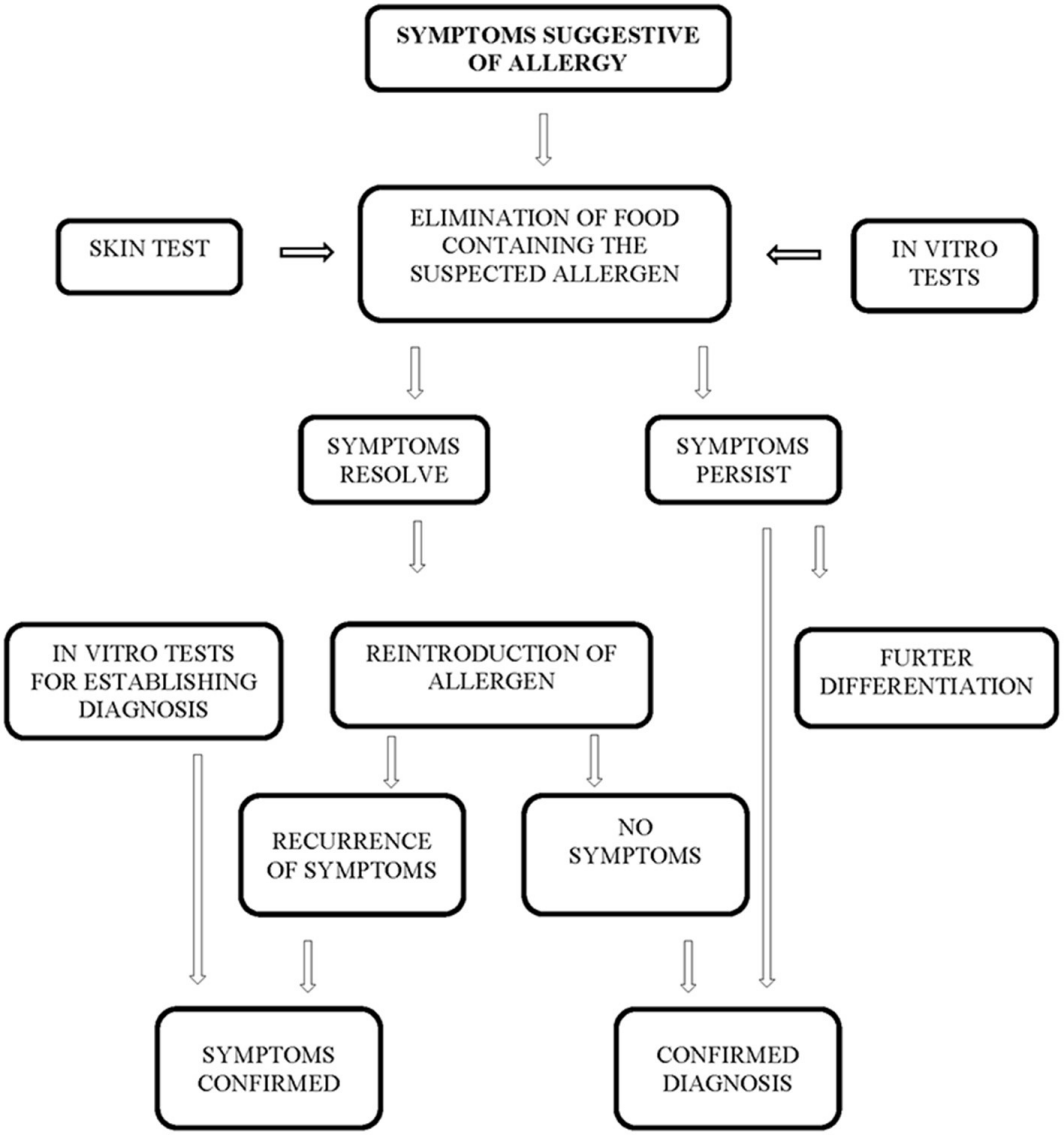
Diagnostic algorithm for establishing the diagnosis of nutritional allergy.

This research was constructed as a longitudinal cohort study. Collection of samples took place between February 2017 and December 2017, at the Pediatric Gastroenterology Clinic of the Balassa János County Hospital. The study population included infants, children and adolescents between 1 and 18 years of age who presented with clinical symptoms suggesting cow's milk protein allergy. Exclusion criteria for enrolment were the diagnosis of inflammatory bowel disease (IBD), coeliac disease (CD), and carbohydrate maldigestion or malabsorption. The diagnosis was established by using the diagnostic algorithm shown in Figure 1 (3, 19).

We collected samples in two stages; however, participants attended the clinic three times. At the first visit, patients with symptoms suggesting cow's milk protein allergy presented to the clinic, where participating children and their parents/legal guardians were given thorough oral and written information about the construction of the study and the methods of sample collection. During this visit the first stool sample was collected, and a proxy questionnaire was filled by the parents in paper-pencil form. The amount of feces was checked, and if the presented amount proved to be insufficient for later diagnostic analysis, submission of a new sample within 24 h was requested. After 1 month of strict dairy elimination diet, we asked parents to reintroduce dairy to the child's diet; the experiences were recorded using a questionnaire. If the child showed symptoms following the consumption of dairy products after a complaint-free period while on elimination diet, a diagnosis of cow's milk protein allergy was established, and the child was enrolled in the final study. Three months of strict elimination diet were followed by a repeated collection of a stool sample in order to monitor the efficacy of the elimination diet with the help of objective parameters; in addition, a third questionnaire was filled by the parents regarding the child's current health status and experiences with the elimination diet.

Out of 55 children, 8 (14.55%) did not meet enrolment criteria, thus biological samples of 47 (male: 57.45%, mean age: 7.36 years, SD: 4.22) children were analyzed. Reasons for exclusion from the study were the following: incomplete filling of questionnaires (*n* = 1, 1.82%) and absence or insufficient quantity of stool samples (*n* = 3, 75.45%); inability to establish a diagnosis of CMPA after 1 month of elimination diet and dairy reintroduction (*n* = 1, 1.8%); voluntary termination of participation (*n* = 3, 5.45%). There was no need for exclusion due to the diagnosis of IBD, CD, and carbohydrate maldigestion or malabsorption.

### Collection of samples

Collection and analysis of the samples were performed following the manufacturer's instructions (Quantum Blue fCAL, Bühlmann Laboratories AG, Schönenbuch, Switzerland). Parents of the participating children received a pre-scripted plastic container equipped with a small spoon for stool collection. Samples were returned to the Pediatric Gastroenterology Clinic within a maximum of 72 h. Samples were stored at−20°C prior to analysis.

### Assessment tools

#### Measurement of stool calprotectin levels

Frozen stool samples were thawed at room temperature. Analysis was performed using Quantum Blue fCAL (Bühlmann Laboratories AG, Schönenbuch, Switzerland) calprotectin rapid tests. One gram of native stool specimen is sufficient for measurement.

The method consists of 3 steps: 1. Sample extraction is performed in the test tube provided with the kit according to instructions. 2. Stool sample is allowed to settle for 10 min, subsequently the supernatant is diluted 1:1.6 extraction buffer; a vortex is used to combine the mixture. The solution is allowed to equilibrate for 5 min at 18–28°C until the next step. 3. There are two approaches to evaluate the sample using the Quantum Blue Reader: CAL 720 and CAL 0.

The first sample is measured with CAL_720 method. 60 μl of the diluted stool sample is added to the test cassette using a pipette, then the'start' button should be pressed. Scanning is automatically started after 12 min (720 s). Since additional samples were collected into pipettes at the same time as the initial specimen, the 12 min incubation period took place outside the machine, hence the CAL 0 method was utilized for the subsequent measurements. Following the pressing of the “start” button, scanning begins immediately. Control measurements follow the methodology described above, using 60 μl of control solution instead of 60 μl of the diluted stool sample. Fecal calprotectin is resistant to proteases and bacterial degradation of pancreatic and intestinal origin, *in vitro* and *in vivo* as well. It is homogenously distributed in stool and remains stable at room temperature for up to 1week, and for several years at−20°C (14, 16, 17, 20).

However, a Dutch study published in 2020 concluded that storage at room temperature for several days can compromise the stability of this protein, thus it is recommended to immediately put samples in a refrigerator (4–6°C) or a freezer (-20°C) (21).

#### Applied questionnaires

The questionnaires–developed by the research team-were completed at the baseline and at the 1st and 2nd follow up. The first questionnaire consisted of questions regarding sociodemographic data, lifestyle-related habits, health status, birth circumstances and CMPA related symptoms. The questionnaire used at the 1st and 2nd follow-up included questions on diet compliance, changes in anthropometric parameters, perceived difficulties to supplement the diet and changes in CMPA related symptoms.

### Statistical analysis

Software from SPSS, Chicago, Illinois, called IBM SPSS 28.0 was used to perform statistical analyses: descriptive statistics were conducted using Wilcoxon rank-sum tests, Mann-Whitney U-tests, paired and independent sample *t*-tests, and multivariate linear regression based on the normality test (Kolmogorov-Smirnov tests). To define predicting factors of calprotectin level we implemented a multivariate linear regression using the stepwise method, where the dependent variable was the calprotectin level and independent variables were age, gender, and symptoms. The level of statistical significance was set at *p* < 0.05.

## Results

### Socio-demographic and clinical data

Forty seven children participated in this study (Table 1). Gender ratio was almost identical, 27 boys and 20 girls were enrolled. The majority of the study population belonged to the kindergarten (31.91 %) and primary school (40.43 %) age groups; and a smaller proportion pertained to the following age groups: toddler (6.38 %), pre-puberty (8.51 %) and puberty (12.77 %). 50% of the children had a sibling with atopy or inflammatory bowel disease; however, these illnesses were detected less frequently in their parents (fathers: 25.53%, mothers: 23.40%).

**Table 1:** Demographic and clinical data of study participants.

**Variables**	**Participants (Study population, *n* = 47) *N* (%)**	**Participants (Strictly following the elimination diet, *n* = 35)** ***N* (%)**
**Gender**		
Male	27 (57.45 %)	19 (54.29 %)
Female	20 (42.55 %)	16 (45.71 %)
**Age group**		
Toddler (1–3 years)	3 (6.38 %)	2 (5.71 %)
Preschool-age (<3–6 years)	15 (31.91 %)	11 (31.43 %)
School-age (<6–11 years)	19 (40.43 %)	12 (34.29 %)
Pre-puberty (<11–14 years)	4 (8.51 %)	4 (11.43 %)
Puberty (<14–18 years)	6 (12.77 %)	6 (17.14 %)
**Family history**		
Mother with atopy or IBD	11 (23.40 %)	8 (22.86 %)
Father with atopy or IBD	12 (25.53 %)	9 (25.71 %)
Sibling with atopy or IBD	16 (45.71 %)	11 (44.00 %)
**Symptoms**		
Gastrointestinal	40 (85.11 %)	30 (85.71 %)
Respiratory	27 (57.45 %)	16 (45.71 %)
Dermatologic	30 (63.83 %)	20 (57.14 %)
Nervous system	21 (44.68 %)	19 (54.29 %)

Enrolled patients most often complained of gastrointestinal symptoms (85.11%), although respiratory (57.45%), skin-related (63.83%) and behavioral (44.68%) symptoms were also frequently reported. In most cases, a concomitance of some of the above-mentioned symptoms was observed.

Regarding the entire study population (*n* = 47), the mean FC level was 73.98 (71.12) μg/g before the strict diet, and 68.11(74.4) μg/g (*p* = 0.331) after the 3-month diet. Since some of the children in the study did not follow a strict diet for the required 3 months, the participants were divided into two groups based on the questionnaires completed by the parents. Comparing the calprotectin levels in these two groups, a significant decrease (*p* = 0.001) was observed after 3-months among those who followed the diet correctly [84.06 (79.48) μg/g vs. 41.11 (34.24) μg/g, respectively]. According to literature FC should be evaluated using adult reference values from about 4 years of age, however we examined calprotectin levels in children under 4 years of age before and after diet and found no difference between the age groups (*p* = 0.518) (22, 23) (Table 2).

**Table 2:** Fecal calprotectin levels of enrolled children.

	**Calprotectin level (**μ**g/g); mean (SD)**		***p*-value**
	**Before diet**	**After diet**	
Study population (*n* = 47)	73.98 (71.12)	68.11 (74.04)	0.331
1–3.99 years old children (*n* = 13)	68.82 (70.79)	73.73 (87.10)	0.859
4–18 years old children (*n* = 34)	75.56 (72.14)	66.39 (70.88)	0.325
Males (*n* = 27)	65.56 (61.16)	71.26 (76.54)	0.851
Females (*n* = 20)	85.35 (82.99)	63.85 (72.26)	0.173
Children following a strict diet (*n* = 35)	84.06 (79.48)	41.11 (34.24)	0.001^**^

** *p < 0.01*.

We also compared the measured results by gender, and we did not find any significant difference in this respect (*p* = 0.545).

According to the results of the two-sample *t* test-among those who followed the diet-the initial calprotectin levels were significantly higher in children with gastrointestinal symptoms (*p* < 0.001) (Table 3).

**Table 3:** Calprotectin levels and symptoms of children following the elimination diet (*n* = 35).

**Symptoms**	**Calprotectin level (**μ**g/g); mean (SD)**		***p*-value**
	**Before diet**	**After diet**	
Gastrointestinal symptoms	90.97 (83.95)	42.97 (36.73)	<0.001^**^
Dermatologic symptoms	60.05 (44.86)	35.30 (13.49)	0.023^*^
Respiratory symptoms	65.31 (48.01)	35.19 (14.80)	0.004^**^
Behavioral symptoms	67.47 (45.60)	33.63 (13.06)	0.001^**^

* *p < 0.05*,

** *p < 0.01*.

As it can be seen in Table 4, significant decrease was measured in calprotectin levels in children following the elimination diet related to abdominal pain (*p* = 0.015), constipation (*p* = 0.008) and diarrhea (*p* = 0.025) as well.

**Table 4:** Gastrointestinal symptoms, calprotectin levels and changes in calprotectin levels in children following the elimination diet (*n* = 35).

**Gastrointestinal symptoms (GI)**	**FC level before diet (μg/g) mean (SD)**	**FC level after diet (μg/g)** ** mean (SD)**	**Change in FC levels mean (SD)**	***p*-value**
Abdominal pain (*n* = 23)	88.52 (90.84)	45.91 (41.46)	54.44 (111.16)	0.015^*^
Vomiting (*n* = 3)	71.67 (21.78)	30.00 (0.00)	41.67 (21.78)	0.109
Belching due to acid reflux (*n* = 8)	59.63 (26.82)	57.63(67.29)	2.67 (92.61)	0.463
Constipation (*n* = 13)	106.77 (97.86)	37.31 (12.96)	75.25 (95.96)	0.008^**^
Diarrhea (*n* = 11)	94.09 (85.59)	39.18 (18.14)	75.50 (95.51)	0.025^*^
No GI symptoms (*n* = 5)	42.60 (9.69)	30.00 (0.00)	15.75 (7.68)	0.044^*^

*FC, fecal calprotectin; GI, gastrointestinal;*

* *p < 0.05*,

** *p < 0.01*.

In our multivariate linear regression model, we examined the variables affecting the follow-up levels of calprotectin. The explanatory power of the regression model is *R*^2^ = 0.169 (*F* = 5.692, *p* = 0.024). The explanatory variables of the model were gender, age, presenting symptom and diet. In the multivariate analysis, only the diet showed a significant effect. (Beta: Regarding the outcome of the follow-up levels of calprotectin, socio-demographic factors and presenting symptoms did not affect the value of the outcome variable.)

## Discussion

A prospective study was intended to assess the effectiveness of a dairy-free diet with an appropriate objective parameter. Based on the assessment of our measuring methods and the questionnaires we conclude that the allergen-induced, moderately elevated FC levels decrease significantly only in children who follow a strict elimination diet; thus, the mentioned biomarker can be an indicator of relief/disappearance of symptoms caused by CMPA.

Earlier it was stated that the most common nutritional allergy in the pediatric population is cow's milk protein allergy (4, 24). As reported in the multicentric survey–which covered every region in Hungary - conducted by the Hungarian Society of Allergology and Clinical Immunology (Magyar Allergológiai Klinikai Immunológiai Társaság – MAKIT), cow's milk is the second most common food allergen in children. The results of this survey are in accordance with international tendencies regarding nutritional allergies (4).

According to a Hungarian research group, the high prevalence of CMPA in children can be explained with a relatively high milk consumption and the immaturity of the immune system (25). In Hungary, during 10 days of public catering in nurseries and kindergarten, 5 and 4 liters of milk should be provided to children, which equals to a daily consumption of 0.5 and 0.4 liters of milk, respectively (26).

As the symptomatology of CMPA is diverse, it may pose a diagnostic challenge even to experienced physicians. Based on the guidelines of the British Society for Allergy and Clinical Immunology, CMPA can manifest as an immediate or as a late-phase allergic reaction. IgE-mediated allergy causes immediate symptoms (anaphylaxis, acute urticaria, vomiting, rhinitis, laryngeal edema, wheezing, acute asthma attack), whereas late-onset symptoms (atopic dermatitis, chronic diarrhea, hematochezia, chronic vomiting, iron deficiency anemia, weight loss) indicate non-IgE mediated allergy; mixed forms can also be often observed symptoms (5, 22, 27).

Measurement of FC is becoming a widely accepted diagnostic method worldwide. Currently it is mostly utilized in establishing the diagnosis of IBD (28, 29) and plays a significant role in differentiation from IBS (irritable bowel syndrome) (30, 31) in both adult and pediatric populations, although some studies in accordance with our research results-suggest that this diagnostic method may be valuable in cases of cow's milk protein allergy. The method of quantification (POCT-Point of Care Test) is a simple, non-invasive, relatively inexpensive, and fast procedure, so it may play a major role in establishing a more accurate diagnosis of CMPA and following-up dietary compliance as well (18).

As it was mentioned above in the study of Belizón et al. 82 infants (1 to 12 months of age) were included, 40 of whom were confirmed to have non-IgE-mediated CMPA. Regarding fecal calprotectin levels, 138 μg/g was considered to be a useful cut-off level to rule out the diagnosis of non-IgE-mediated CMPA. In their experience, FC is not a suitable diagnostic method for predicting a clinical response to an elimination diet (12).

In contrast, a study in Turkey has drawn a completely opposite conclusion. Beser et al. examined the effect of a strict milk-free diet using a FC test on 32 newly diagnosed CMPA children (10.16 ± 8.57 month of age). The results of the study confirm that the strict diet showed a significant decrease in FC values (*p* < 0.001) (11).

In our current research, children under 1 year of age were excluded due to their naturally higher stool calprotectin levels as pointed out often in previous publications (22, 32). According to Fagerberg et al. (23) a cut-off level of 50 μg/g for FC–as determined for adult population-can be used in children from 4 years of age (22). In the study of Davidson et al. (33) the upper limit of the normal FC level for children is 62 mg / kg between 4 and 17.9 years of age. In our research, a calprotectin level of 50 μg/g was regarded as normal for a pediatric population.

In our case, no significant difference in calprotectin levels was observed in children under 4 years of age [pre-diet calprotectin level 68.82 (70.79) μg/g, post-diet level 73.73 (87.10) μg/g, *p* = 0.859] and children older than 4 years [re-diet calprotectin level 75.56 (72.14) μg/g, post-diet 66.39 (70.88) μg/g, *p* = 0.325], therefore the two age groups were not examined separately.

The results of our study-similar to Beser's (11)-also showed a significant decrease in FC levels in the group of strict dieters [before diet mean: 84,057 (79.48) μg/g, after diet mean: 41,114 (34.24) μg/g, *p* < 0.001], so elimination/alleviation of clinical symptoms is expected with a strict elimination diet. Therefore, we propose measuring FC levels to monitor the extent of intestinal inflammation caused by the allergen.

## Data availability statement

The raw data supporting the conclusions of this article will be made available by the authors, without undue reservation.

## Ethics statement

The studies involving human participants were reviewed and approved by Institutional Research Ethics Committee of the Balassa János Teaching Hospital Regional and Institutional Research Ethics Committee of the University of Pécs. Written informed consent to participate in this study was provided by the participants' legal guardian/next of kin.

## Author contributions

DL-E: design of the study, analysis and interpretation of data, and drafting the manuscript. GT: conception and design of the study and revising the manuscript. VE: drafting and revising the manuscript. PS: interpretation of the data and critical revising the manuscript. VP, KA, PT, and KE: interpretation of data and revising the manuscript. AM and KF: analysis and interpretation of the data and revising the manuscript. All authors fulfill the requirements of authorship, intellectually contributed to the study, read, and approved the final manuscript.

## Funding

This research was supported by the Gedeon Richter's Talentum Foundation (1103 Budapest, Gyömroi Str.19-21.), Thematic Excellence Program 2021 Health Sub-program of the Ministry for Innovation and Technology in Hungary, within the framework of the EGA-16 project of the University of Pécs, the National Research, Development and Innovation Office (KF 132834), by the ÚNKP-21-5 New National Excellence Program of the Ministry for Innovation and Technology from the source of the National Research, Development and Innovation Fund (ÚNKP-21-5-PTE-1341), and by the János Bolyai Research Scholarship of the Hungarian Academy of Sciences (BO/00317/21). The funding sources did not have any role in the study design; in collection, analysis, and interpretation of data, or in writing and submitting this manuscript.

## Conflict of interest

The authors declare that the research was conducted in the absence of any commercial or financial relationships that could be construed as a potential conflict of interest.

## Publisher's note

All claims expressed in this article are solely those of the authors and do not necessarily represent those of their affiliated organizations, or those of the publisher, the editors and the reviewers. Any product that may be evaluated in this article, or claim that may be made by its manufacturer, is not guaranteed or endorsed by the publisher.
